# Morph-specific seasonal variation of aggressive behaviour in a polymorphic lizard species

**DOI:** 10.7717/peerj.10268

**Published:** 2020-11-20

**Authors:** Alan Jioele Coladonato, Marco Mangiacotti, Stefano Scali, Marco A. L. Zuffi, Carlotta Pasquariello, Cristian Matellini, Simone Buratti, Mara Battaiola, Roberto Sacchi

**Affiliations:** 1 Department of Earth and Environmental Sciences, University of Pavia, Pavia, Italy; 2 Museo di Storia Naturale di Milano, Milano, Italy; 3 Museo di Storia Naturale dell’Università di Pisa, Calci, Italy

**Keywords:** Colour polymorphism, Aggressive behaviour, *Podarcis muralis*, Seasonal variation, Alternative strategies, Mirror test

## Abstract

The persistence of colour polymorphism (CP) within a given population is generally associated with the coexistence of alternative reproductive strategies, each one involving specific trade-offs among behavioural, morphological, physiological, and other life histories. Common wall lizard (*Podarcis muralis*), is a medium-sized diurnal lizard, showing CP in three main colours (yellow, white, and red) on throat and belly, and a morph-specific pattern for both immunocompetence and seasonal variation of T levels. Yellow males show low stamina with high plasma T levels at the beginning of the season, while white males show high stamina with a higher plasma T levels at the end of the season. We hypothesised the presence of two strategies: a risky one, characterised by high aggressiveness played by yellow-morph, and a conservative one by white morph with low aggressiveness. Thus, we tested the aggressive response to conspecifics of yellow and white morphs using a mirror inserted into their cage, mimicking an intrusion of a stranger in their territories, throughout the breeding season (from April to July, 117 trials). We considered three types of aggressive response, with different levels of aggressiveness: (i) bite against the image reflected in the mirror, (ii) seconds spent by the individuals into the half mirrored cage, and (iii) number of times the lizard entered the half mirrored cage. We also considered the number of tongue flicking as explorative behaviour variable. All lizards were tested after a period of acclimatisation to the captivity conditions. Results demonstrate that yellow males showed a higher aggressive response in the early season and a decrease aggressive response towards the end, whereas white males showed an opposite pattern.

## Introduction

Species exhibiting colour polymorphism (CP) are excellent model systems to understand evolutionary processes. Following Huxley’s (1955) definition CP is ‘the presence of two or more genetically distinct forms which co-occur in both time and space, with the rarest form too common to be solely attributed to recurrent mutation’. This is a widespread phenomenon with extensive documentation in taxa through the Animal Kingdom, in both invertebrates and vertebrates (Mckinnon & Pierotti, 2010; Wellenreuther, Svensson & Hansson, 2014). CP can be maintained by natural and sexual selection, it is often genetically correlated, and the process which generates and maintains it can affect speciation and/or extinction rates, either positively or negatively (Gray & Mckinnon, 2006; Hugall & Stuart-fox, 2012). CP provides opportunities to explore how sexual selection works and how evolution maintains the coexistence of different phenotypes, since morphs represent an easily identifiable genotype-phenotype correspondence (Mckinnon & Pierotti, 2010). Within a given population, CP is generally associated with the coexistence of alternative reproductive strategies, which are modulated by complex interactions among environmental pressures (e.g. social interactions and individual density), each one involving specific trade-offs among behaviour, physiological, and other life-history traits (Roulin & Bize, 2007). Therefore, a colour morph represents an alternative, locally adapted optimum, that is an optimal combination of the traits involved in the trade-offs, which equally optimise the fitness (Sinervo & Lively, 1996; Svensson, Sinervo & Comendant, 2001; Roulin & Bize, 2007; Sacchi et al., 2007a; Andrade et al., 2019).

Trade-offs are usually thought in terms of resource allocation, where the allocation of limited resources to one trait has negative consequences for the other traits requiring the same resource (Zera & Harshman, 2001). Trade-offs involving the immune system are particularly interesting since the ability to deal with parasites and diseases has severe consequences on the fitness any individual may achieve, but also entails substantial costs for them (Lochmiller & Deerenberg, 2000; Demas, 2004; Cox, Peaden & Cox, 2015). A trade-off involving immunity is the one stated by the Immunocompetence Handicap Hypothesis (ICHH, Folstad & Karter, 1992), that assumes (and predicts) an inverse relationship between plasma testosterone (T) level and immune function. In lizards, the focus of our study, testosterone increases territoriality, home-range size and its quality (Marler & Moore, 1988; DeNardo & Sinervo, 1994; Fox, 1983), also affecting body shape and fertility in a variety of vertebrates (Cox et al., 2005; Ketterson & Nolan, 1992; Oliveira, 2004; Hau, 2007). Variation in plasma T level is related to aggressive behaviour in many species of vertebrates (Rose, Holaday & Bernstein, 1971; Rada, Kellner & Winslow, 1976; Greenberg & Crews, 1983; Harding, 1983). Aggressive behaviour leads to higher territorialism, which results in more females and more resources. Thus, high T levels would ultimately favour reproductive success (Negro et al., 2010; Mokkonen et al., 2012). However, maintaining high T levels has costs, non-only through the increased predatory risk associated to the T-driven behaviour, but also in terms of immune functions decrease, which favours parasite infections, and eventually degrades survival (Olsson et al., 2000; Klukowski & Nelson, 2001; Cox & John-Adler, 2007). Evidence in favour of ICHH has been found over different vertebrate taxa (Roberts, Buchanan & Evans, 2004; Mills et al., 2008; Foo et al., 2016).

Among vertebrates, reptiles represent a good model to investigate the evolution and maintenance of CP, particularly lizards (Gray & Mckinnon, 2006). In this clade, many species maintain marked CP at the population level and some underlying mechanisms have been already hypothesised and tested (Sinervo & Lively, 1996; Thompson & Moore, 1991; Thompson, Moore & Moore, 1993; Hews et al., 1997; Zamudio & Sinervo, 2000; Sinervo & Zamudio, 2001; Sacchi et al., 2007a; Huyghe et al., 2009; Runemark, Gabirot & Svensson, 2011). Notably, in males, the different breeding strategies often involve the modulation of aggressiveness against contending conspecifics according to the colour morph per se or to the morph combination of the focal and contending male (Sinervo & Lively, 1996; Hover, 1985; Thompson & Moore, 1991; Thompson, Moore & Moore, 1993). In many cases, the aggression level displayed and the contest outcome may be predicted by the colour morphs, irrespective of other asymmetries in size, residency, or prior experience (Sinervo & Lively, 1996; Hover, 1985; Thompson & Moore, 1991; Thompson, Moore & Moore, 1993). For instance, in the ornate tree lizard (*Urosaurus ornatus*), green males are more likely to dominate orange ones despite their smaller size (Hover, 1985); in the common blotched lizard (*Uta stansburiana*), orange males are highly aggressive over all other colour morphs (Sinervo & Lively, 1996), and in the painted dragon (*Ctenophorus pictus*), red males have a higher probability of winning the contest against yellow ones (Healey, Uller & Olsson, 2007). Further, in the above species, the more aggressive morph also showed higher absolute T levels (Knapp & Moore, 1997; Sinervo et al., 2000; Olsson et al., 2007). However, in other species of lizards, the link between CP and aggressiveness is less straightforward, and colouration appears to influence only the fight outcome between unfamiliar opponents (Stuart-Fox & Johnston, 2005; Sacchi et al., 2009).

On the immune side, the suppressive effect of testosterone is well-documented in many lizard species. An artificial increase in T levels decreases cell-mediated immunity (Olsson et al., 2000; Belliure, Smith & Sorci, 2004; Oppliger et al., 2004) and has been related to an increase of ectoparasites load and to hematological parameters variation (Puerta et al., 1996; Salvador et al., 1996, 1997; Veiga et al., 1998; Klukowski & Nelson, 2001; Pollock, Vredevoe & Taylor, 2012). In the light of these findings and since the alternative aggressive strategies constitute different fitness optima (Sinervo & Lively, 1996; Sinervo et al., 2000), we can hypothesise that the the trade-off stated by ICHH could play a role on the maintenance of the CP, where each morph is associated with a different behaviour strategy which consequently entails a connected immune response (Sacchi et al., 2009, 2017b).

The Common wall lizard (*Podarcis muralis*) is a medium-sized diurnal lizard (50–70 mm adult snout-vent length, SVL) that shows three main colour morph in both sexes, white, yellow, and red, on throat and belly (Sacchi et al., 2007b). The colourations develop from the second year of life (Cheylan, 1988) and the role of CP in social communication was widely studied in the last decades (Sacchi et al., 2009, 2015, 2017a, 2017b; Scali et al., 2013; Pellitteri-Rosa et al., 2014; Abalos et al., 2016; Pérez i de Lanuza, Carretero & Font, 2017; Mangiacotti et al., 2019a, 2019b). A morph-specific pattern in the immune response has been demonstrated (Sacchi et al., 2007a) and the same occured in plasma T levels throughout the breeding season (Sacchi et al., 2017b). These results show that yellow males are immunosuppressed compared to the other morphs, and bear higher plasma T levels at the beginning of the reproductive season (April), and lower at the end (July). These data suggest that the yellow males play a different strategy compared to the other colour morphs, that is yellow males invest more energy in aggressive interactions and intrasexual-competition at the beginning of the breeding season, at the expense of better stamina (as stated by ICHH); consequently, they are expected not to be able to maintain the needed aggressive level also in the late season (Sacchi et al., 2017b). However, a previous study found no morphs difference in aggressiveness (Sacchi et al., 2009), although there was a non-significant trend for red to lose when paired against yellow or white males (Abalos et al., 2016). Given these mixed results, and since the time of the breeding season was not considered, we conducted a study to examine if aggression varied in a morph-specific manner across the season. This question is motivated by documented morph-specific differences plasma T level and immune function, across the breeding season. In this study, we measured the aggressive response of male Common wall lizard morphs throughout the breeding season, to test the hypothesis of a morph-specific strategy, explicitly accounting for the time-dimension where the strategy is expected to be played.

## Materials and Methods

### Lizards collection and housing

During spring and early summer 2018, we collected 117 adult males (69 white morph and 48 yellow morph, SVL > 50 mm) (Sacchi et al., 2007b) of Common wall lizard (*Podarcis muralis*) by noose in Pavia (Northern Italy, Lombardy). In order to track the whole reproductive season, captures were conducted every week from April to July, trying to balance colour morphs within each session, and at least two individuals each morph and session were collected. Since white and red males show the same T level seasonal pattern (Sacchi et al., 2017b), and red males occur at low frequency in Pavia populations (Sacchi et al., 2007b), we focused only on white and yellow morphs.

Within 2 hours of capture, the lizards were transferred to the University of Pavia, at the Department of Earth and Environmental Sciences, and housed in individuals Plexiglas cages (20 × 30 × 20 cm) with the four walls covered with white papersheets (to avoid visual disturbance during behavioural tests, see below). Each cage was provided with shelter, water ad libitum and lizards were fed with one mealworm (*Tenebrio molitor*) per day. Each lizard was measured (to the nearest 0.1 mm using a calliper) for snout-vent length (SVL) after the trial to reduce handling stress; the body mass (accuracy ± 0.1 g) was recorded at the capture time and after the trial, to assess that the housing protocol had no effect (*P* = 0.06). Mean SVL was 63.4 ± 3.0 mm (range 57.5–72.0 mm) for the yellow morph and 63.0 ± 3.5 mm (range 55.8–71.0 mm) for the white morph. There was no difference in size between morphs (two samples *t*-test, *t* = 0.69, df = 115, *P* = 0.49). The housing room was maintained between 15 and 32 °C, simulating the temperature range observed in Pavia in late spring and early summer (Karger et al., 2017), and natural daylight was guaranteed. Trials started after an acclimation period of at least 7 days, to allow individuals to consider the cage as their own territory (Mangiacotti et al., 2019b). All lizards were released, healthy, at their captured sites, within a maximum of 2 weeks from their capture.

### Experimental setting

To measure the aggressive response, we used a mirror test to mimick an intrusion of a stranger in the individual’s territory (the cage). This method allows removing the size and motivation effects by showing to the tested individual an image with the same behaviour, size, and motivation (Sacchi et al., 2009; Scali et al., 2019, 2020). After acclimation, lizards were tested in their own cage after removing the water bowl. To avoid visual disturbance during the experiments, the four sides of the cage were externally covered with white paper. The experimental protocol consisted in: (i) heating the lizard in its cage for 5 min with a 75 W halogen infrared lamp positioned 40 cm above the cage; (ii) inserting the mirror, covered by a plastic septum, in the cage wall opposite to the shelter; (iii) after 3 more minutes, removing the septum and recording lizard behaviour, using a webcam (Microsoft LifeCam HD 3000) positioned above the cage and connected to a laptop. Recording duration was set to 15 min and started at the first exploratory movements (i.e. tongue flicking, head movements towards the mirror, etc.). Videos were managed by Free2X software v1.0.0.1 (freely available at http://www.free2x.com/webcam-recorder/), setting quality to 800 × 600 pixels and 15 fps (*frames per second*). The trials were run between 10 a.m. and 2 p.m., and the order of morphs was randomised to remove any potential effect of day-time. At the end of each trial we measured the body temperature of lizards with a handheld infra-red thermometer (Lafayette TRP-39, Lafayette Instrument Co., Lafayette, IN, USA; sensitivity: 0.1 °C; precision: ±2%)

### Response variables

We processed all videos using BORIS open-source software (Behavioral Observation Research Interactive Software, available at www.boris.unito.it, Friard & Gamba, 2016), which allowed us to extract from each video an ethogram consisting in four response variables: (i) the total number of bites against the reflected image (Bites); (ii) the time spent in the half mirrored cage (Time, in s); (iii) the number of times each individual entered the half mirrored cages (Nmirror); (iv) the ratio of the number of tongue flicks to Time (RTF). While the first three variables were considered proxies for different levels of aggressive behaviour, the fourth one evaluated the basal explorative behaviour of each individual when facing a potential contestant (Sacchi et al., 2020, unpublished data). In particular, we considered Bites as the maximum level of aggressiveness, such as direct aggression to the ‘rival’; Nmirror as the interest in facing the ‘rival’, and Time as the interest for the ‘rival’, since the time spent in the half mirrored cage would have been the same as the one spent in the other half if the mirrored image did not elicit the interest of the focal male: the longer the time, the larger is the interest (Sacchi et al., 2020 unpublished data). All variables were weakly correlated with each other (Spearman correlation coefficient: ${\left| {{r_{Spearman}}} \right|_{max}}$ = 0.31).

### Statistical analysis

Time, Nmirror, and RTF assumed a normal distribution (One-sample Kolmogorov-Smirnov test, all *P* values larger than 0.05), while Bites showed a Poisson-like distribution with overdispersion (sd/mean = 34), and zero inflation. Thus, we ran Zero-Inflated Negative Binomial Regression (ZINB) for Bites and a Linear Model (LM) for Time, Nmirror, and RTF. Julian date (hereafter Day), morphs and their interaction entered the model as fixed effects, in order to assess whether the response variables varied over the season but with different patterns between morphs. We also added SVL and body temperature as a fixed effect to control for possible confounding effects due to age and individual activity. All analyses were done in R 3.6.2 (R Core Team, 2019) using the package glmmABDM (Fournier et al., 2012), and otherwise stated, data reported are means ± standard errors. The study was performed following the European and Italian laws on animal use in scientific research, and all the protocols have been authorised by the Italian Environmental Ministry (Aut. Prot. PNM0002154.03-02-2016, valid for the 3 years 2016–2018).

## Results

Lizards approached the mirror in 116 out of 117 trials (99.1%), and bit the mirrored image in 82% of cases. The time spent in the half mirrored cage ranged from 0 to 1,198 s, being on average 786 s, and lizards entered the half portion of the cage hosting the mirror on average 6.2 times each trial (range 0–20, Table1). Nmirror was on average 6 ± 0.4 for white males (range 1–19) and 6.3 ± 0.6 for yellow males (0–20); the mean for Time was 805 ± 33 s (range 54–1,199) for white males and 759 ± 46 s (0–1,174) for yellow males. RTF was identical in the two morphs (Table 1). Finally, the most frequent number of bites to the mirror (mode and interquartile distance) was 13 (11–64) and 5 (1–35) in white and yellow males respectively (Table 1).

**Table 1:** Descriptive statistics of the results of the behavioural experiments.

	White	Yellow	Total
Bites*	13 (11–64) (0–116)	5 (1–35) (0–92)	8 (3–50) (0–116)
NMirror	6 ± 0.4 (1–19)	6.3 ± 0.6 (0–20)	6.2 ± 0.35 (0–20)
Time	805 ± 33 (54–1,199)	759 ± 46 (0–1,174)	786 ± 27 (0–1,199)
Ratio of Tongue Flicking	0.09 ± 0.01 (0–0.28)	0.09 ± 0.01 (0–0.23)	0.09 ± 0.01 (0–0.28)

**Note:**

Mean (min–max) values of each response variable. Asterisk (*) is for Poisson distributed variables and the mode is indicated with the first and third quartiles. For all variables, the range is indicated (min–max) above.

**Figure 1:**
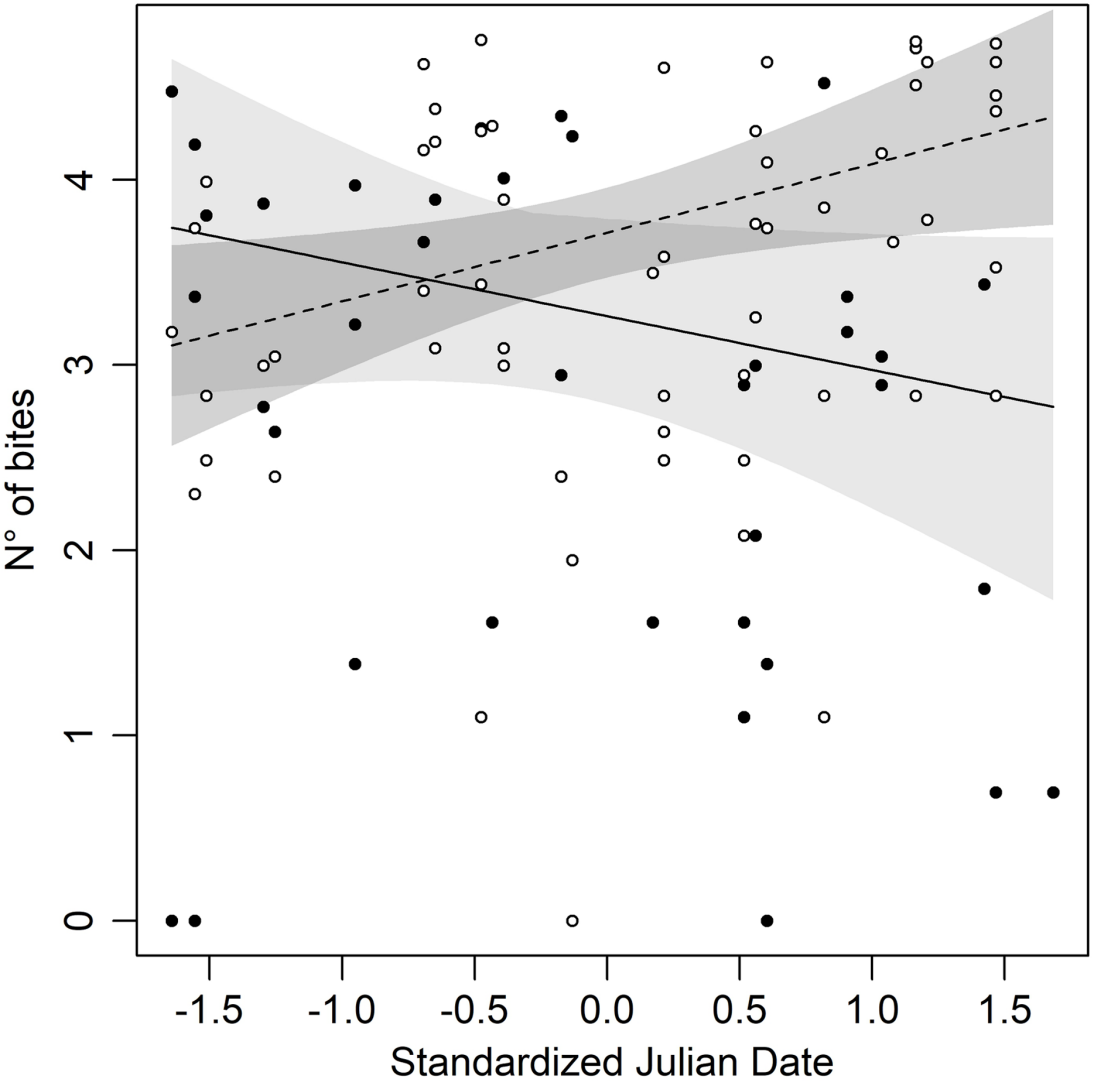
Number of bites against the mirror throughout the season in both morphs of Common wall lizard. Yellow morph: black dot = observed values, dashed line = predicted line, light grey area = 95% confidence interval; white morph: white dot = observed values, continuous line = predicted line, dark grey area = 95% confidence intervals.

The statistical analysis showed that Bites significantly varied depending on the Day (then, with the season), morphs, and their interaction (Table 2), suggesting that the aggressive behaviour changes during the season with a morph specific pattern. A significant increase in bites has been observed in white males (Day: β = 0.37 ± 0.16; *P* = 0.02), whereas the opposite pattern has been observed in yellow males, although not in a significant statistical way (Day: β = −0.29 ± 0.16; *P* = 0.07). Specifically, yellow males at the beginning of the season had higher aggression than white males, but as the season went on this difference disappeared until a switch of the aggressive behaviour in the two morphs did occur. Consequently, late in the season, white males were more aggressive to the mirror than yellow males (Fig. 1). Finally, our results show no significant effect of temperature and size for Bites (Table 2).

**Table 2:** Results of statistical analysis.

	Df	*X²*	*P*
*Bites*			
Day	1	5.68	**0.02**
Morph	1	4.78	**0.03**
SVL	1	0.58	0.45
Temperature	1	1.38	0.24
Day:morph	1	9.31	**0.002**
*Time*			
Day	1	0.73	0.39
Morph	1	1.35	0.25
SVL	1	2.06	0.15
Temperature	1	2.13	0.14
Day:morph	1	0.30	0.59
*Nmirror*			
Day	1	0.07	0.80
Morph	1	0.33	0.56
SVL	1	0.07	0.79
Temperature	1	0.76	0.38
Day:morph	1	1.31	0.25
*Ratio of tongue flicking*			
Day	1	0.01	0.77
Morph	1	0.12	0.73
SVL	1	0.10	0.75
Temperature	1	1.90	0.17
Day:morph	1	0.08	0.78

**Note:**

Effects of the experiment date, morphs, SVL, final temperature, and interaction Day × morph on Bites, Time, Nmirror, and Ratio of Tongue Flicking in males of *Podarcis muralis*. Significant *P* values are reported in bold.

The statistical analysis performed for Time, Nmirror and RTF did not show any statistically significant effect of both Day and colour morph (Table 2): individuals explored the space in the same way regardless of size, season, temperature, or morphs.

## Discussion

In this study we measured the variation of aggressive response during the breeding season in *P. muralis* males using a mirror, mimicking an intrusion of a stranger in the lizards’ territory. Our experiment clearly showed that yellow males were more aggressive (i.e. bit their mirrored image) than white males early in the season, but this difference reversed as the season progressed. The lack of a significant difference in the explorative behaviour, that is RTF, between morphs along the season, supports the idea that seasonal variation in aggression was not due to the experimental setting. The results of this study agree with the hypothesis that aggressive behaviour is a morph specific trait, and it is displayed with different patterns in the two morph according to the specific breeding strategies they adopt. However, only the highest expression of aggressive behaviour (i.e. Bites) showed this seasonal, morph specific trend (increases in whites and decreases in yellows). Indeed, we did not find any significant difference in both Nmirror and Time, which measured a lower-level aggressive expression. Nmirror and Time represent threatening attitudes at distance, which did not imply a direct physical interaction with the opponent, and consequently no risk of injuries. So, the costs of those displays directly affecting the ICHH trade-off are probably low, and, therefore, are not relevant in shaping the morphs’ alternative strategies. By contrast, overt aggression, that is Bites, has potentially high costs in terms of both injuries and stress. Among all the variables, our result shows that only ‘Bite’ varied seasonally, and in a morph-specific manner, an this supports the hypothesis that they modulate the ICHH trade-off in two different ways (i.e. with two alternative strategies), depending on the costs they are willing to sustain during aggressive interactions with conspecific males.

Male Common wall lizards can recognise individuals belonging to their morph (Pérez i de Lanuza et al., 2018) and they show higher aggression towards individuals belonging to their same morph (Scali et al., 2020). They also show greater aggressiveness in a familiar context (i.e. their territory) with a high subjective resource value than in unfamiliar context (Sacchi et al., 2009; Sacchi et al., 2020 unpublished data). Having said that, our experimental setting reflect the most stimulating context, regard with Resource-Holding-Potential and morph-specific aggressive pattern (Sacchi et al., 2009; Scali et al., 2020; Sacchi et al., 2020 unpublished data), which allows to consider the obtained results as the maximum possible aggressive response in a given period. These results agree with the morph-specific seasonal pattern in plasma T levels found in Sacchi et al. (2017b) and support our initial hypothesis which envisaged the existence of a more aggressive strategy (played by yellow males) as opposed to a more conservative one (played by white males). Increased aggressive behaviour in the early part of the season means more clashes among individuals, but at the cost of lower long-term survival due to higher predatory risk and a lower immune response (Marler & Moore, 1988; Sacchi et al., 2009) to the benefits of those who choose the ‘conservative strategy’. Since aggressive behaviour has an inverse correlation with the immune response, as stated by ICHH, we can say that these alternative strategies come out by a trade-off between two contrasting needs: the investment in territorial aggression on one hand and a longer survival on the other.

Adopting a different strategy depending on morphs can help individuals to recognise the strategy of rivals and modulate their own. Many species of vertebrates show an aggressive morph-specific response: an example is given by cichlids fish *Metriaclima mbenjii* where males direct more aggression towards similarly coloured opponents (Van Doorn, Dieckmann & Weissing, 2004; Seehausen & Schluter, 2004; Dijkstra et al., 2006, 2007, 2008, 2010; Pauers et al., 2008) and similar results happened in the polymorphic sparrow, *Zonotrichia albicollis* (Horton, Hauber & Maney, 2012). It is also recurrent in reptiles: in *Urosaurus ornatus* males the manipulation of colour triggers aggression against opponents (Hover, 1985); experiments performed with colour-manipulated models of *Ctenophorus decresii* showed a higher aggressive behaviour during homomorphic contexts (Yewers, Pryke & Stuart-Fox, 2016). Former studies concerning aggressive morph-specific response as a mechanism underlying polymorphism maintenance in Common wall lizard showed contrasting results. Sacchi et al. (2009) did not find any correlations between the aggressive strategy and individual’s morph; instead, Abalos et al. (2016) found a lower fighting ability in red males, but this could be due to the size of black patches. Finally, Scali et al. (2020) demonstrated that *P. muralis* shows a greater aggressive response if the contender belongs to its own morph. The results of our experiments clearly show that aggression is modulated over time by morphs, and it is important to consider the time window when carrying out this type of behavioural experiment because it would provide different seasonal results based on the morph. For example, if you carry out behavioural experiments too close to the inversion point (Fig. 1) you will not notice aggressive response differences between morphs, resulting in a false negative.

High aggression leads to a greater chance of winning the encounters and consequently a greater chance of breeding. The existence of alternative strategies could be due to the opportunity of males to invest in different clutches. Previous studies on reproductive biology of lacertid lizards, and in particular of *P. muralis*, indicate that female lizards show two peaks of deposition in late April and late May (Sacchi et al., 2012; Galeotti et al., 2013). Moreover, material for yolk production of the first clutch is mainly derived from fat reserves stored before hibernation, whereas subsequent clutches are influenced by the available resources in the current season (Braña, Gonzalez & Barahona, 1992). So, the seasonal aggressiveness modulation in male morphs may synchronise with female deposition timing, suggesting that yellow males may aim at the first clutch (based on female fat reserves), whereas white males at the following ones (depending on resource availability along the season).

## Conclusions

Our study shows that seasonal pattern of variation in aggression in male Common wall lizards is morph specific and in accordance with the prediction of the trade-off promoted by the ICHH. According to it, white and yellow males of this species could adopt two alternative strategies involving different investment in aggression and immunity function leading to a riskier strategy (yellow males) and a more conservative one (white males). Yellow males tend to be more aggressive at the beginning of the season, while white males are able to maintain an overall higher aggressiveness along the season. The different phenology of the aggressive behaviour between male morphs may help to explain the persistence and coexistence of different morphs in a population.

## Supplemental Information

10.7717/peerj.10268/supp-1 Supplemental Information 1 Detailed sample number per category. Samples size of Common wall lizards’ males for each morph collected throughout breeding season. Dates reporter refer to collection day in the field and not the date of experiments.

10.7717/peerj.10268/supp-2 Supplemental Information 2 Biometrics and variables used for statistical analysis.

## Additional Information and Declarations

### Data Availability

The following information was supplied regarding data availability: All data that we used for statistical analysis are available in the Supplemental Files.
