# Supplementary material for: Morph-specific seasonal variation of aggressive behaviour in a polymorphic lizard species
Source: PeerJ. 2020 Nov 20;8:e10268. doi: 10.7717/peerj.10268 (PMC7682419; doi:10.7717/peerj.10268)
Supplement: Supplemental Information 1 — Samples size of Common wall lizards’ males for each morph collected throughout breeding season. Dates reporter refer to collection day in the field and not the date of experiments. [file peerj-08-10268-s001.docx]

|  | 1-15 April | 16-30 April | 1-15 May | 16-31 May | 1-15 June | 16-30 June |  | Total |
| --- | --- | --- | --- | --- | --- | --- | --- | --- |
| White | 7 | 4 | 19 | 14 | 16 | 9 |  | 69 |
| Yellow | 7 | 8 | 10 | 9 | 10 | 4 |  | 48 |

**Table 3.** Samples size of Common wall lizards’ males for each morph collected throughout breeding season. Dates reporter refer to collection day in the field and not the date of experiments.
